# Efficacy and safety of biliary stenting combined with ^125^I seed implantation for the treatment of advanced extrahepatic cholangiocarcinoma

**DOI:** 10.3389/fsurg.2025.1608312

**Published:** 2025-09-22

**Authors:** Cai Cheng, Bo Wang, Chunmu Miao, Shengwei Li

**Affiliations:** ^1^ Department of Hepatobiliary Surgery, The Second Affiliated Hospital of Chongqing Medical University, Chongqing, China; ^2^ Department of General Surgery, The Thirteenth People’s Hospital of Chongqing (Chongqing Geriatrics Hospital), Chongqing, China

**Keywords:** extrahepatic cholangiocarcinoma, ^125^I seed implantation, biliary stenting, stent patency time, postoperative survival

## Abstract

**Background:**

The implantation of ^125^I seed is expected to improve the prognosis of patients undergoing stent placement for advanced extrahepatic cholangiocarcinoma (eCCA), but its efficacy and safety remain unclear.

**Methods:**

Forty advanced eCCA patients who received percutaneous transhepatic biliary stenting (PTBS) (control group) and 40 PTBS combined with ^125^I seed implantation (^125^I group) were retrospectively analyzed. Changes in serum biochemical indicators and tumor markers as well as the occurrence of complications were observed in the two groups, and the durations of stent patency and survival were compared.

**Results:**

The general information and preoperative baseline data did not significantly differ between the two groups (*P* < 0.05). Regardless of whether PTBS was combined with ^125^I seed implantation, the ALT/AST levels of patients after operation were significantly lower, jaundice was relieved. And the improvements in postoperative liver function and jaundice in patients in ^125^I group were better than those in control group. In addition, tumor markers in the two groups decreased significantly, and the decrease was more significant in patients in ^125^I group. There was no significant difference in the total complication rate between the two groups. The stent patency time and overall survival of the patients in the ^125^I group were longer than those in control group.

**Conclusion:**

Biliary stenting combined with ^125^I seed implantation is a safe and effective treatment for patients with advanced eCCA, and it is superior to biliary stenting alone in improving liver function and prolonging the duration of stent patency and survival time.

## 1 Introduction

Cholangiocarcinoma (CCA) is a malignant tumor that occurs in the epithelial cells of the bile duct, with the second-degree bile duct used as an anatomical marker (1). Intrahepatic cholangiocarcinoma is located at the proximal segment of the secondary bile duct, while extrahepatic cholangiocarcinoma (eCCA) is found from the primary bile duct to the bile-pancreatic ampulla. eCCA encompasses perihilar cholangiocarcinoma (pCCA) and distal cholangiocarcinoma (dCCA). pCCA arises from the principle bile duct to the confluence of the hepatic duct and cystic duct, whereas dCCA extends from the cystic duct to the bile-pancreatic ampulla (2).

eCCA mainly manifests with bile duct obstruction, for which radical surgical resection is still the only curative means (3, 4). The 5-year survival rates of pCCA and dCCA patients after radical surgical resection are 20%–30% and 18%–43%, respectively (5–8). For eCCA patients who are inoperative, unwilling to undergo surgery, or who are in poor systemic condition and cannot tolerate surgery, the palliative treatment is mainly biliary drainage, which includes external biliary drainage and internal biliary drainage. The internal biliary drainage are in line with physiology to avoid body fluid loss (9).

The implantation of self-expandable metallic stent (SEMS) is the main means of bile-enteral drainage. However, the stent itself cannot stop tumor growth, and more than 50% of SEMSs are reblocked after 6 months due to tumor overgrowth, leading to rehospitalization and re-intervention and greatly reducing patients' quality of life (10–12). How to inhibit tumor growth, prolong the patency time of patients with biliary stents and overall survival (OS) and improve the quality of life of patients are major clinical problems at present.

^125^I particles are a low-energy radiation source, with the persistent emission of low-energy gamma rays capable of inducing damage to the DNA double strands of tumor cells (13, 14). This process effectively eradicates tumor cells across various stages of cell division, inhibits tumor proliferation, and facilitates apoptosis. The effective radiation radius of ^125^I particles is 1.7 cm, which delivers a high dose to tumor tissues and causes low damage to surrounding tissues (15); therefore, they have been widely used to treat malignant tumors. Especially, ^125^I particles are widely used in solid tumors, such as liver cancer, prostate cancer, lung cancer, and pancreatic cancer, and have achieved good efficacy (16–19). In recent years, researchers have applied radioactive stents combined with ^125^I seeds in the treatment of eCCA. However, studies of ^125^I seed implantation combined with biliary stenting in patients with extrahepatic BDT are relatively rare and heterogeneous (20, 21).

Therefore, in this study, we aimed to conduct a retrospective analysis to evaluate the potential enhancement in efficacy of biliary stenting when used in conjunction with ^125^I particles.

## 2 Materials and methods

### 2.1 Study population

Patients with advanced eCCA who received PTBS (control group) or PTBS combined with ^125^I seed implantation (^125^I group) at our center between January 2018 and January 2022 were retrospectively analyzed. The study was approved by the Ethics Committee of the Second Affiliated Hospital of Chongqing Medical University and was in compliance with the Declaration of Helsinki. All patients signed informed consent before treatment.

The inclusion criteria were as follows: (1) Patients diagnosed clinically with eCCA; (2) Patients whose tumor were not resectable or who were unwilling to undergo surgical resection; (3) PS score: 0–1. The exclusion criteria included the following: (1) Patients with distant organ metastasis; (2) The full course of follow-up could not be performed; (3) Patients were complicated with severe portal hypertension and had a previous history of upper gastrointestinal bleeding or severe hypersplenism; (4) Significant abnormalities in coagulation function or liver and kidney failure were detected.

### 2.2 Technical methods

**Figure 1:**
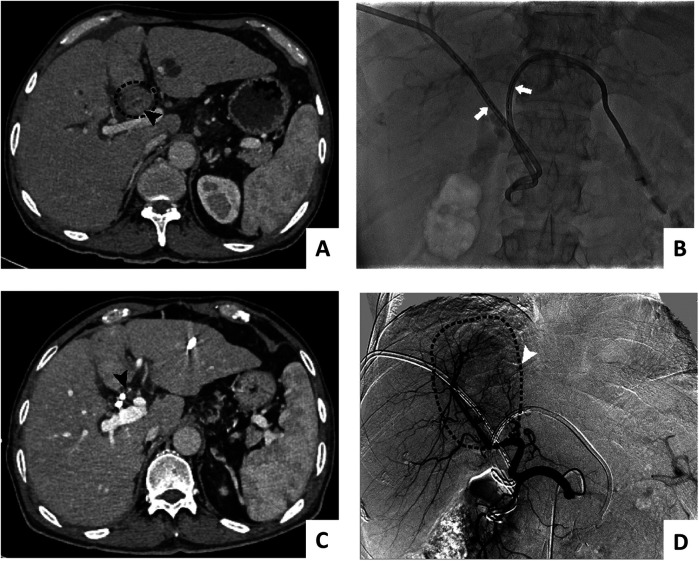
Procedural and follow-up imaging of ^125^I stent-seed assembly: pre-implantation **(A)**, successful stent-seed implantation **(B)**, 1-month follow-up imaging (projection 1) **(C)**, 1-month follow-up imaging (projection 2) **(D)**

1. Percutaneous transhepatic biliary drainage (PTBD): The target bile duct for puncture and drainage is selected in combination with enhanced CT or enhanced MRI, and percutaneous transhepatic external bile duct drainage was performed under the guidance of ultrasound combined with x-ray (diameter less than or equal to 6 mm).
2. Biliary stenting: The selection of 8 mm or 10 mm stents was guided by the diameter of target bile ducts, site-specific obstruction characteristics, and physician's clinical judgment per standard clinical protocols. The guidewire was implanted in the intrahepatic bile duct through the PTBD channel, the sheath was implanted along the guidewire, the guidewire cooperated with the catheter through the bile duct tumor site and entered the distal bile duct, and the biliary metal was implanted along the guidewire. The stent covered the bile duct obstruction 2 cm beyond the proximal end and the distal end of the stenosis.
3. Biliary stenting combined with ^125^I seed implantation: a stent suitable for the diameter of the bile duct and the length of the obstructed segment and the long 6F sheath were placed side by side over the bile duct tumor; the stent was released first, and then the homemade ^125^I particles (PTBD tube plastic carrying ^125^I particles) were placed in parallel. The catheter with a soft inner core was trimmed to a suitable length for the bile duct tumor (the particle segment was 1 cm longer than the upper and lower ends of the bile duct tumor) and was delivered to the location of the bile duct tumor through the long sheath. Procedural and follow-up imaging of ^125^I stent-seed assembly was shown in Figure 1.

### 2.3 Data collection

A patient information database was established, which included patient age, sex, tumor location, and preoperative serological test results, including total bilirubin (TBIL), direct bilirubin (DBIL), alanine aminotransferase (ALT), aspartate aminotransferase (AST), alkaline phosphatase (ALP), glutamyl transpeptidase (GGT), albumin (ALB), C-reactive protein (CRP), white blood cell (WBC), neutrophil count (NC), lymphocyte count (LC), platelet count (PLT), prothrombin time international normalized ratio (PT-INR), carbohydrate antigen 19-9 (CA19-9), and carcinoembryonic antigen (CEA) levels.

All patients were followed up until July 2024, or the patient died. The follow-up data included TBIL, DBIL, ALT, AST, ALP, ALB, CA19-9, and CEA levels.

### 2.4 Statistical analysis

SPSS software version 27 was used for the statistical analysis. Count data are expressed as percentages and frequencies and were compared via the Pearson chi-square test or the Pearson chi-square test with continuity correction. Continuous variables are expressed as the means ± standard deviations or medians with ranges, and comparisons were performed via t tests or Wilcoxon tests. OS and cumulative primary stent patency time were evaluated via Kaplan‒Meier analysis and compared via the log-rank test. A *p* value of less than 0.05 was considered statistically significant.

## 3 Results

### 3.1 Baseline information

**Figure 2:**
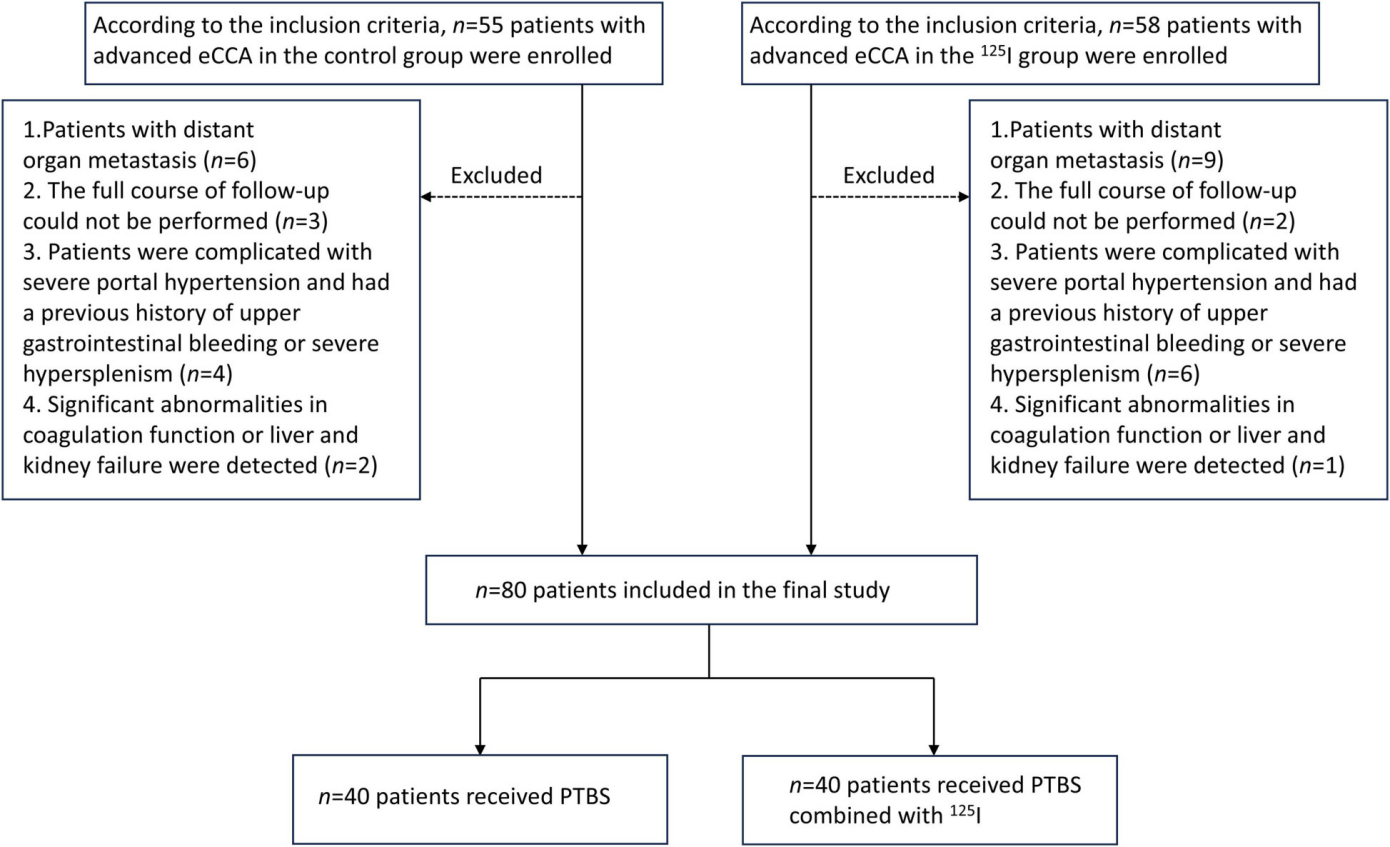
Patient enrollment flowchart. Schematic illustrating participant selection for the comparative study of ^125^I stent efficacy in eCCA patients undergoing PTBS. eCCA: extrahepatic cholangiocarcinoma; PTBS: percutaneous transhepatic biliary stenting.

Forty advanced eCCA patients who received percutaneous transhepatic biliary stenting (PTBS) alone (control group) or 40 patients who received PTBS combined with ^125^I seed implantation (^125^I group) were included (Figure 2). The baseline clinical characteristics of these 80 patients are summarized in Table 1. In the control group, there were 17 males (42.5%) and 23 females (57.5%), and the mean age was 69.20 ± 6.90 years. Among them, 19 patients were were diagnosed with pCCA, and 21 patients were were diagnosed with dCCA. In the 125I group, there were 21 males (52.5%) and 19 females (47.5%), and the mean age was 69.33 ± 7.13 years. Among them, 13 patients were were were diagnosed with pCCA, and 27 patients were were diagnosed with dCCA. There were no significant differences in preoperative liver function, jaundice, or tumor markers between the two groups of patients (*P* > 0.05). A biliary stent or biliary stent combined with ^125^I particles was successfully implanted in both groups of patients, the biliary tract was unobstructed after operation, and the surgical success rate was 100%.

**Table 1:** Comparison of baseline information.

Variables	Control group (*n* = 40)	^125^I group (*n* = 40)	*P* value
Age (years)	69.00 (55, 80)	70.00 (53, 78)	0.962
Male (%)	42.5	52.5	0.37
Location (pCCA/dCCA)	19/21	13/27	0.171
CA19-9 (ng/ml)	966.22 ± 534.64	1,116.70 ± 585.42	0.234
CEA (ng/ml)	5.47 ± 2.27	6.35 ± 2.78	0.125
ALT (U/L)	158.95 55.14	152.85 51.82	0.612
AST (U/L)	155.20 ± 50.43	146.20 ± 58.67	0.464
ALP (U/L)	586.78 ± 227.96	548.93 ± 194.54	0.427
GGT (U/L)	1,102.93 ± 543.51	1,020.40 ± 491.89	0.479
TBIL (µmol/L)	159.18 ± 84.01	178.88 ± 93.84	0.326
DBIL (µmol/L)	146.56 ± 79.48	153.32 ± 77.13	0.7
ALB (g/L)	36.71 ± 3.28	36.57 ± 3.07	0.844
PT-INR	1.1 [0.9,1.3]	1.1 [0.7,2.1]	0.263
NC (10^9^/L)	6.49 ± 1.96	6.68 ± 2.21	0.68
LC (10^9^/L)	1.2 ± 0.42	1.19 ± 0.26	0.896
PLT (10^9^/L)	371.08 ± 109.46	370.78 ± 128.46	0.991
CRP (mg/L)	30.72 ± 12.3	27.34 ± 13.57	0.246

ALB, albumin; ALP, alkaline phosphatase; ALT, alanine aminotransferase; AST, aspartate aminotransferase; CA19-9, carbohydrate antigen 19-9; CCA, cholangiocarcinoma; CEA, carcinoembryonic antigen; CRP, C-reactive protein; DBIL, direct bilirubin; GGT, glutamyl transpeptidase; LC, lymphocyte count; NC, neutrophil count; PLT, platelet count; PT-INR, prothrombin time international normalized ratio; TBIL, total bilirubin; WBC, white blood cell.

### 3.2 Postoperative liver function and jaundice

**Figure 3:**
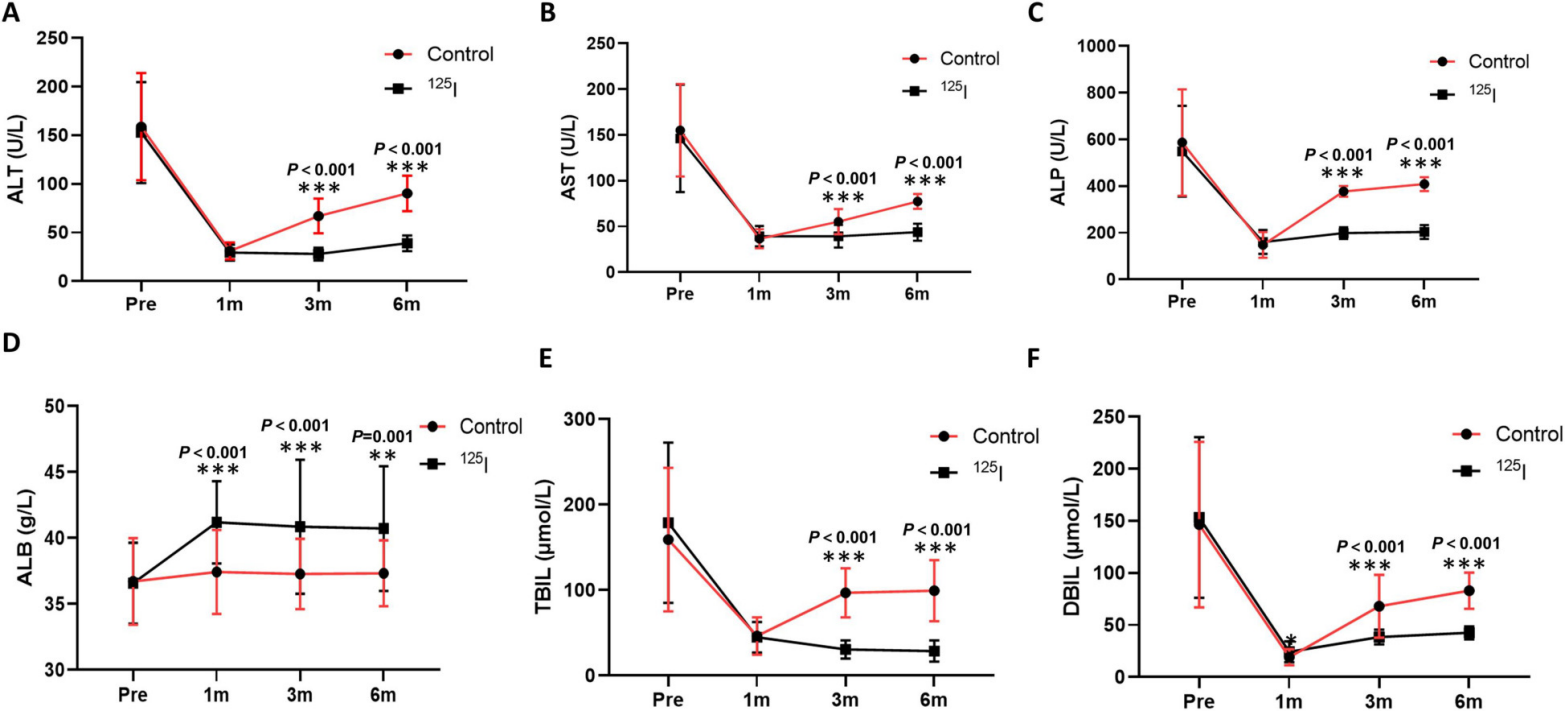
Comparison of ALT **(A)**, AST **(B)**, ALP **(C)**, ALB **(D)**, TBIL **(E)**, and DBIL **(F)** levels between the two groups after operation. *P < 0.05, **P < 0.01, ***P < 0.001. ALB, albumin; ALP, alkaline phosphatase; ALT, alanine aminotransferase; AST, aspartate aminotransferase; DBIL, direct bilirubin; TBIL, total bilirubin.

Compared with the preoperative level, liver function in both groups was significantly improved after operation. This improvement is evidenced by significant reductions in ALT, AST, and ALP levels at 1, 3, and 6 months following the operation (Figures 3A–C), alongside a significant increase in ALB levels (Figure 3D). There was no significant difference between the ^125^I group and the control group in the ALT, AST, and ALP levels at 1 month, but there was a significant difference between 3 and 6 months. The ALB levels at 1, 3, and 6 months in the 125I group were greater than those in the control group. The symptoms of jaundice in the two groups were significantly relieved in both groups, which was reflected in the significant decreases in TBIL and DBIL (Figures 3E,F). In addition, the TBIL and DBIL levels in the ^125^I group were significantly lower than those in the control group at 3 and 6 months. These results suggest that both biliary stenting alone and biliary biliary stenting combined with ^125^I seed implantation can improve postoperative liver function and reduce obstructive jaundice and that biliary stenting combined with ^125^I seed implantation are superior to biliary stenting alone.

### 3.3 Postoperative tumor marker levels

**Figure 4:**
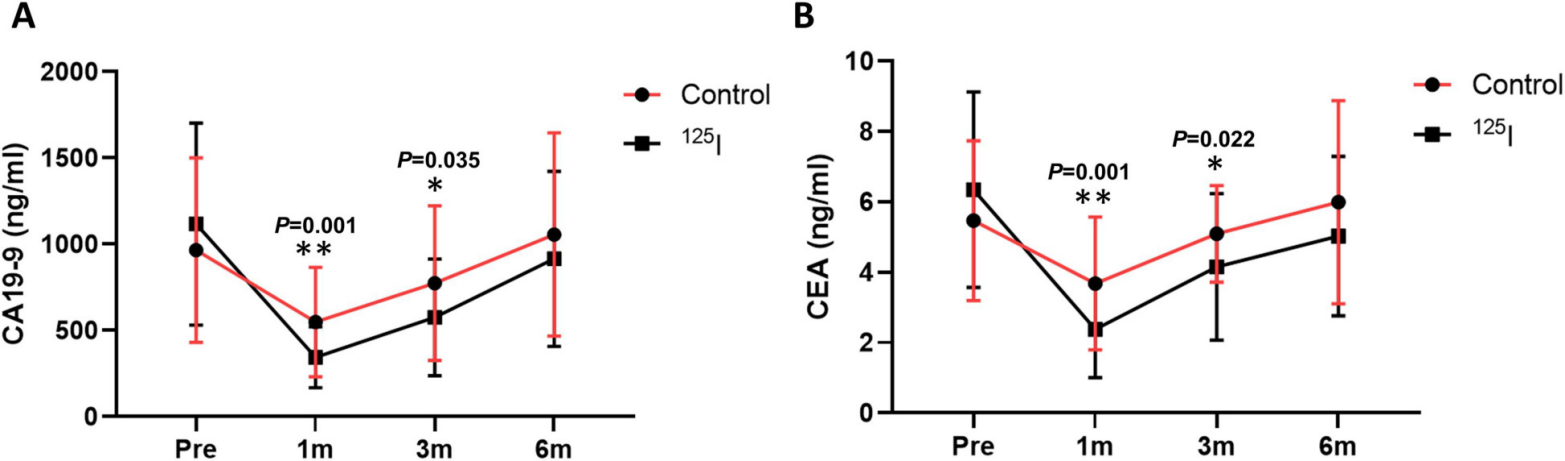
Comparison of CA19-9 **(A)** and CEA **(B)** levels between the two groups after operation. *P < 0.05, **P < 0.01, ***P < 0.001. CA19-9, carbohydrate antigen 19-9; CEA, carcinoembryonic antigen.

CA19-9 and CEA levels after operation are shown in Table 2. Compared with the preoperative CEA and CA19-9 levals, the follow-up values of the patients with increased CEA and CA19-9 values after 1 month of treatment all decreased to different degrees (Figures 4A,B). In addition, the CEA and CA19-9 levels of the ^125^I group at 1 and 3 months after operation were lower than those of the control group.

**Table 2:** CA19-9 and CEA levels in two groups after operation.

Outcomes	Control group	125I group	*P* value
CA-199			
Pre-treatment	966.22 ± 534.64	1,116.7 ± 585.42	0.234
Post-1 month	548.42 ± 317.45	344.87 ± 176.73	0.001
Post-3 months	773.82 ± 448.50	576.53 ± 338.34	0.035
Post-6 months	1,056.38 ± 589.15	915.49 ± 507.56	0.322
CEA			
Pre-treatment	5.47 ± 2.27	6.35 ± 2.78	0.125
Post-1 month	3.68 ± 1.89	2.38 ± 1.38	0.001
Post-3 months	5.09 ± 1.37	4.15 ± 2.08	0.022
Post-6 months	5.99 ± 2.89	5.03 ± 2.26	0.150

CA19-9, carbohydrate antigen 19-9; CEA, carcinoembryonic antigen.

### 3.4 Postoperative complications

As shown in Table 3, the overall incidence of early complications was 17.5% (14 patients), with 12.5% (5 patients) and 22.5% (9 patients) in the control group and the ^125^I group, respectively. There was no significant difference in the total incidence between the two groups. Specifically, 4 patients in the control group and the ^125^I group both developed cholangitis, and no patients in the two groups developed bile duct bleeding. Gastrointestinal complications (nausea and vomiting) occurred in 1 patient in the control group and 6 patients in the ^125^I group, respectively, but there was no significant difference between the two groups. The symptoms of nausea and vomiting were mild and were gradually relieved.

**Table 3:** Comparison of the incidence of postoperative complications.

Group	Cholangitis	Biliary bleeding	Nausea and vomiting	Total complication
Control group	4 (10%)	0 (0%)	1 (2.5%)	5 (12.5%)
^125^I group	4 (10%)	0 (0%)	6 (15%)	9 (22.5%)
Total	8 (10%)	0 (0%)	7 (8.8%)	14 (17.5%)
*P* value	1	/	0.113	0.239

### 3.5 Bile duct patency and overall survival

**Figure 5:**
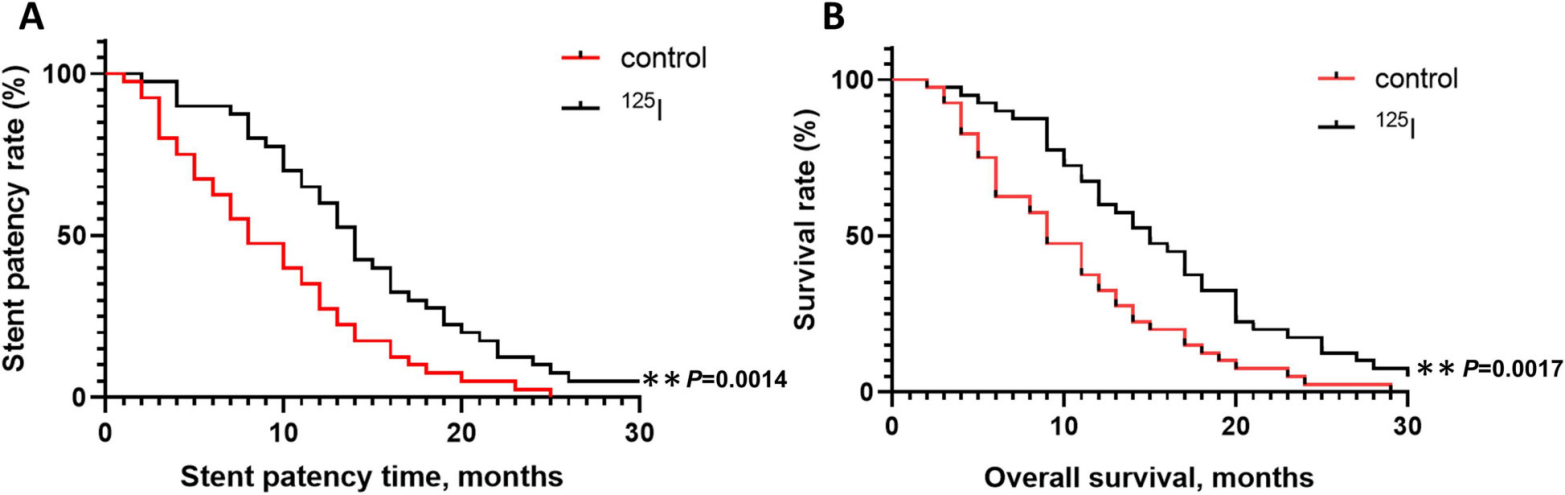
Stent patency rates of the ^125^I and control groups **(A)** overall survival rates of the ^125^I and control groups **(B)** *P < 0.05, **P < 0.01, ***P < 0.001.

The patency conditions of the stents in the two groups are shown in Figure 5A. The cumulative patency rates of the control group at 3, 6, and 12 months were 92.5%, 67.5% and 35%, respectively, and the median patency time was 8 months. The cumulative patency rates of the ^125^I group at 3, 6, and 12 months were 97.5%, 90% and 65%, respectively, and the median patency time was 14 months. The hazard ratio (HR) for the ^125^I group compared to the control group was 0.446 (95% CI: 0.271, 0.732), indicating a statistically significant difference and significantly longer patency time in the ^125^I group. The postoperative survival data are shown in Figure 5B. The cumulative survival rates of the control group at 3, 6, and 12 months were 97.5%, 75% and 37.5%, respectively, and the median survival time was 9 months. The cumulative survival rates of the ^125^I group at 3, 6, and 12 months were 97.5%, 92.5% and 67.5%, respectively, and the median survival time was 15 months. The HR for the ^125^I group compared to the control group was 0.452 (95% CI: 0.275, 0.742), demonstrating a statistically significant difference and significantly longer survival time in the ^125^I group.

## 4 Discussion

Self-expandable metal stent is a common choice for enteral drainage in eCCA patients, but its effectiveness is not satisfactory. In this study, we included 40 patients in the control group who underwent biliary stent implantation and 40 patients in the ^125^I group who underwent biliary stent implantation combined with ^125^I particles implantation. The results confirmed that, compared with the control group, the ^125^I group had significantly improved postoperative liver function, relieved symptoms of obstructive jaundice, reduced tumor marker levels, and prolonged stent patency time and OS without serious complications.

The most common symptom of eCCA is obstructive jaundice, and biliary drainage is an indispensable treatment technique for unresectable eCCA patients with obstructive jaundice. Endoscopic biliary stent placement was introduced in the 1980s; owing to its minimal invasiveness and practicability, it is now the preferred procedure for the palliative treatment of most eCCA patients. Although there are various types of stents, metal stents are recommended for unresectable cases because they have longer patency time than plastic stents do. In recent years, chemotherapy, immunotherapy and other methods prolong the survival of patients (22). However, a new difficulty arises: the bile duct will still be blocked again, and the patient will experience a recurrence of jaundice in a short period of time, which seriously affects the survival and quality of life of the patient (23).

Stent re-occlusion fundamentally arises from a cascade effect of tumor activity, foreign body reaction, and bile flow dysregulation. Neoplastic cells progressively invade the lumen through longitudinal overgrowth at stent margins or transverse ingrowth through stent interstices. Concurrently, the stent itself induces chronic inflammation and fibroblast activation, driving granulomatous hyperplasia and collagen-deposition stenosis. These processes collectively cause bile stagnation, promoting supersaturation and precipitation of bilirubin/cholesterol into sludge matrices that entrap calcium salts and bacterial biofilms, ultimately forming obstructive calcium bilirubinate stones. This self-reinforcing cycle, wherein obstruction exacerbates sludge formation, sludge accumulation accelerates infection and hyperplasia, and hyperplastic tissue further constricts the lumen, demands integrated therapeutic strategies targeting all pathological axes simultaneously. It is precisely this multidimensional challenge that makes biliary stenting combined with radioactive seed implantation a rational solution: the stent restores immediate drainage patency while the radionuclide's localized irradiation concurrently suppresses tumor proliferation, mitigates inflammatory hyperplasia, and delays sludge formation through continuous biological modulation (24, 25).

At present, ^125^I particles have been confirmed to be effective and safe in the treatment of portal vein tumor thrombi (26, 27). Because eCCA is discovered in the advanced stage and clinical cases are rare, the application of ^125^I seed implantation combined with biliary stenting in advanced eCCA is rare at home and abroad, the research directions are new, and there is currently no unified treatment guidelines. On the basis of the above studies combined with our previous clinical observations, we speculated that biliary stent with brachytherapy of ^125^I seed implantation may result in better treatment outcomes for patients with eCCA and provide a standardized treatment option for patients with advanced eCCA.

In this study, we collected the clinical data of 80 patients with advanced eCCA combined with obstructive jaundice. The results showed both groups effectively reduced obstructive jaundice, improved liver function, and reduced postoperative tumor marker levels in the short term. However, over time, in the stent alone group, tumor growth again caused lumen stenosis or blockage; the stent patency rates at 3 and 6 months after operation were 92.5% and 67.5%, respectively. Additionally, there was a rapid increase in ALT, AST, and bilirubin. In contrast, in the biliary stent combined with ^125^I seed implantation group, because ^125^I can perform long-term and low-dose radiation on tumor tissues, stent patency can be maintained for a longer time. The stent patency rates at 3 and 6 months, respectively, were 97.5%, 90%. Moreover, the ALT, AST, and bilirubin levels at 3 and 6 months were significantly lower than those in the biliary stent alone group, showing a gradual upward trend. The same situation was also observed for postoperative tumor marker levels. These advantages led to longer survival in the biliary stent combined with ^125^I seed implantation group, with a median survival of 15 months, which was significantly greater than that of the biliary stent alone group (9 months). With respect to the long-term efficacy of treatment for advanced eCCA, the implantation of a biliary stent combined with ^125^I seeds was superior to internal biliary stent implantation alone. Although more postoperative complications were observed in the biliary stent with ^125^I seed implantation group, the main complications were gastrointestinal symptoms (nausea, vomiting, etc.), which were relieved within a short time after operation and did not have a significant impact on patient's life.

This study has several limitations. Especially, our study was limited to a single-center, retrospective study, but the baseline levels of the included two groups of patients were not different, which makes up for this limitation to a certain extent.

## 5 Conclusion

In summary, the therapeutic efficacy of biliary stenting combined with ^125^I particles implantation for the treatment of advanced eCCA is superior to that of stenting alone. This combined approach effectively relieves biliary obstruction, facilitates the restoration of liver function, and extends both biliary tract patency and patient survival duration. Therefore, the integration of biliary stenting with 125I particle implantation represents a highly promising strategy for patients with advanced eCCA.

## Data Availability

The original contributions presented in the study are included in the article/Supplementary Material, further inquiries can be directed to the corresponding author.
